# Supplementary material for: Importin-β modulates the permeability of the nuclear pore complex in a Ran-dependent manner
Source: eLife. 2015 Mar 6;4:e04052. doi: 10.7554/eLife.04052 (PMC4375889; doi:10.7554/eLife.04052)
Supplement: Supplementary file 1. — DOI: http://dx.doi.org/10.7554/eLife.04052.022 [file elife04052s001.docx]

### Supplementary Table 1 – Constructs

| **Plasmid** | **Description** | **Resistance** | **Source** |
| --- | --- | --- | --- |
| **Importin**-**β** | His_6_ – Importin-β | Kanamycin | Adam *et al*. [[1](#_ENREF_1)] |
| **Ran** | His_6_ – Ran | Kanamycin | Kalab *et al*. [[2](#_ENREF_2)] |
| **RanQ69L** | His_6_ – RanQ69L | Kanamycin | Kalab *et al.* [2] |
| **NTF2** | His_6_ – NTF2 | Ampicillin | Paschal *et al*. [[3](#_ENREF_3)] |
| **Impβ−YFP** | His_6_ – YFP – Importin-β | Kanamycin | This study |
| **Impβ−mCherry** | His_6_ – mCherry – Importin-β | Kanamycin | This study |
| **Impβ−mEos2** | His_6_ – mEos2 – Importin-β | Kanamycin | This study |
| **Transportin-1** | Transportin-1 – His_6_ | Kanamycin | Görlich *et al.* [4] |
| **Transportin-1–GFP** | GST – GFP – Transportin-1 | Ampicillin | Kose *et al.* [5] |
| **GFP_1_** | His_6_ – GFP | Kanamycin | This study |
| **GFP_2_** | His_6_ – GFP – GFP | Kanamycin | This study |
| **GFP_3_** | His_6_ – GFP – GFP – GFP | Kanamycin | This study |
| **SnpIBB – 2xGFP** | His_6_ – SnpIBB – GFP – GFP | Kanamycin | This study |
| **ImpαIBB – Cerulean** | His_6_ – ImpαIBB – Cerulean – Biotin | Ampicillin | This study |
| **Nup153FG** | GST – (TEV) – Nup153(874-1475) – His_6_ | Ampicillin | Lim *et al.* [[6](#_ENREF_4)] |

1. Adam, S.A., R.S. Marr, and L. Gerace, *Nuclear protein import in permeabilized mammalian cells requires soluble cytoplasmic factors.* J Cell Biol, 1990. **111**(3): p. 807-16.

2. Kalab, P., K. Weis, and R. Heald, *Visualization of a Ran-GTP gradient in interphase and mitotic Xenopus egg extracts.* Science, 2002. **295**(5564): p. 2452-6.

3. Paschal, B.M. and L. Gerace, *Identification of Ntf2, a Cytosolic Factor for Nuclear Import That Interacts with Nuclear-Pore Complex Protein P62.* J Cell Biol, 1995. **129**(4): p. 925-937.

4. Görlich, D., et al., *A novel class of RanGTP binding proteins.* J Cell Biol, 1997. **138**(1): p. 65-80.

5. Kose, S., et al. *The 70-kD heat shock cognate protein (hsc70) facilitates the nuclear export of the import receptors.* J Cell Biol, 2005. **171**(1): p19-25.

6. Lim, R.Y., et al., *Flexible phenylalanine-glycine nucleoporins as entropic barriers to nucleocytoplasmic transport.* PNAS, 2006. **103**(25): p. 9512-7.
